# Whole Genome DNA Methylation Variations in Mammary Gland Tissues from Holstein Cattle Producing Milk with Various Fat and Protein Contents

**DOI:** 10.3390/genes12111727

**Published:** 2021-10-28

**Authors:** Mengqi Wang, Nathalie Bissonnette, Pier-Luc Dudemaine, Xin Zhao, Eveline M. Ibeagha-Awemu

**Affiliations:** 1 Sherbrooke Research and Development Centre, Agriculture and Agri-Food Canada, Sherbrooke, QC J1M 0C8, Canada; mengqi.wang@agr.gc.ca (M.W.); nathalie.bissonnette@agr.gc.ca (N.B.); pier-luc.dudemaine@agr.gc.ca (P.-L.D.); 2 Department of Animal Science, McGill University, Ste-Anne-De-Bellevue, QC H9X 3V9, Canada; xin.zhao@mcgill.ca

**Keywords:** DNA methylation, whole genome bisulfite sequencing, mammary gland tissue, Holstein cattle, milk fat content, milk protein content, rRNA, QTL

## Abstract

Milk fat and protein contents are among key elements of milk quality, and they are attracting more attention in response to consumers′ demand for high-quality dairy products. To investigate the potential regulatory roles of DNA methylation underlying milk component yield, whole genome bisulfite sequencing was employed to profile the global DNA methylation patterns of mammary gland tissues from 17 Canada Holstein cows with various milk fat and protein contents. A total of 706, 2420 and 1645 differentially methylated CpG sites (DMCs) were found between high vs. low milk fat (HMF vs. LMF), high vs. low milk protein (HMP vs. LMP), and high vs. low milk fat and protein (HMFP vs. LMFP) groups, respectively (*q* value < 0.1). Twenty-seven, 56 and 67 genes harboring DMCs in gene regions (denoted DMC genes) were identified for HMF vs. LMF, HMP vs. LMP and HMFP vs. LMFP, respectively. DMC genes from HMP vs. LMP and HMFP vs. LMFP comparisons were significantly overrepresented in GO terms related to aerobic electron transport chain and/or mitochondrial ATP (adenosine triphosphate) synthesis coupled electron transport. A total of 83 (HMF vs. LMF), 708 (HMP vs. LMP) and 408 (HMFP vs. LMFP) DMCs were co-located with 87, 147 and 158 quantitative trait loci (QTL) for milk component and yield traits, respectively. In conclusion, the identified methylation changes are potentially involved in the regulation of milk fat and protein yields, as well as the variation in reported co-located QTLs.

## 1. Introduction

Genetic selection for milk production up to the end of the last century principally focused on increasing milk yield, but with the dawn of the 21st century, the focus has broadened to include milk quality, health, welfare, fitness and environmental sustainability traits [1]. In addition to consumer preferences, attention on improving milk component yield, especially milk fat/fatty acids and protein yields, has been precipitated by its influence on the nutritional, physical and flavor properties of milk products. Over the past decades, implementation of genetic selection led to increased genetic gain for milk fat and protein content [2,3,4,5,6]. In recent times, implementation of genomic selection has revolutionized dairy cow breeding with increased gains realized in all traits being targeted for selection, including fat and protein yields [7]. Genomic selection, however, relies on sequence variations such as single nucleotide polymorphisms (SNP) that do not account for all the phenotypic variance in traits, thereby opening up the possibility that other processes such as epigenetic mechanisms also contribute to phenotypic variation in livestock traits [8,9].

Epigenetic mechanisms including DNA methylation alteration have been identified as important regulatory mechanisms of mammary gland development and health [10,11,12,13]. Associations between DNA methylation alterations and milk production in cattle have been reported [13,14,15] leading to the prediction of DNA methylation as an insightful molecular mechanism underlying the phenotypic changes of milk production traits [9,15]. Specifically, genome-wide DNA methylation changes were reported between dairy cattle with different milk yields, revealing potential association between DNA methylation and milk yield [16,17]. Recently, negative association between global DNA methylation rates and milk protein yield were identified in lactating dairy cows, revealing that DNA methylation may play a role in milk protein production [16]. Moreover, unusual DNA methylation around the STAT5-binding enhancer in the αS1-casein promoter negatively regulated αS1-casein synthesis in milk during lactation [18,19,20]. Furthermore, increased DNA methylation of some lipid-related genes, such as ACACA, SCD, FASN, and PPARG, regulated their gene expression and thereby affected fatty acid synthesis and milk fat content of goat milk [21,22].

Despite recent research attention to explore the potential influence of DNA methylation on cattle production and health traits, the contribution of DNA methylation to milk production, especially the content of milk fat and protein, is still poorly understood. Therefore, this study profiled the genome-wide DNA methylation patterns of mammary gland tissues from Holstein cattle with various milk fat and protein content as well as examined potential association between DNA methylation alterations and milk composition.

## 2. Materials and Methods

### 2.1. Experimental Animals and Sample Collection

Experimental cows were from the dairy herd of the Sherbrooke Research and Development Centre (SRDC) of Agriculture and Agri-Food Canada. Animals were managed according to routine farm management practices. A composite milk sample (30 mL) was collected once monthly from all lactating cows (*N* = 96) over a period of 12 months and the components quantified to identify cows producing milk with very high or very low fat and protein contents for four consecutive months.

### 2.2. Milk Component Analysis

Milk component analysis including test day milk fat and protein yields was determined with MilkoScan FT 6000 Series mid-range infrared Fourier Transform Infra-Red (FTIR) based spectrometers by Lactanet (lactanet.ca; Ste-Anne de Bellevue, QC, Canada). Test day milk fat and protein yields were determined by multiplying the respective percentages with the total test day milk production. 

Cows with consistently highest milk fat (HMF, MF > 4.80%, *N* = 7) or protein (HMP, MP > 3.60%, *N* = 5) content over a four-month period consecutively were selected. Similarly, cows with consistently lowest milk fat (LMF, MF ≤ 3.7%, *N* = 5) or protein (LMP, MP ≤ 3.15%, *N* = 7) contents over the same period were selected. Among them, some cows were qualified in more than one group, including three cows producing milk with both high fat and high protein contents (HMFP, MF > 4.80% and MP > 3.6%) as well as three cows producing milk with low fat and low protein contents (LMFP, MF ≤ 3.7% and MP ≤ 3.15%). The parities of cows ranged from 1 to 7 and day in milk ranged from 48 to 352 (Table 1). Mammary gland tissue samples or biopsies were aseptically collected from the 17 cows through surgery by a professional veterinarian and following standard procedures at the SRDC dairy barn. Mammary gland tissues were cut into small pieces, immediately snap frozen in liquid nitrogen and stored at −80 °C until used. 

Animal use procedures were approved in accordance with the guidelines of the Canadian Council on Animal Care, and ethical approval to conduct the study was provided by the Animal Care and Ethics Committee of Agriculture and Agri-Food Canada (approval #571).

### 2.3. DNA Isolation, WGBS Library Construction and Sequencing

Genomic DNA was isolated from mammary gland tissues using DNeasy^®^ Blood & Tissue kit (Qiagen Inc., Toronto, ON, Canada) following manufacturer’s instructions. The Qubit dsDNA high-sensitivity (HS) assay (Invitrogen by ThermoFisher Scientific, Mississauga, ON, Canada) was used to quantify DNA. Genomic DNA was sheared into short fragments (about 200–500 bp), and size selected using SPRIselect beads (Beckman Coulter, Mississauga, ON, Canada). Fragmented DNA was bisulfite converted using EZ DNA Methylation Lightning Kit (Zymo Research, Tustin, CA, USA) according to the manufacturer’s recommendations. Five hundred ng bisulfite converted DNA per sample was used for library preparation with the Accel-NGS Methyl-Seq DNA library kit (Swift Biosciences, Ann Arbor, MI, USA) following manufacturer′s instructions. Then, dual-indexed adapters were added followed by 6 cycles of polymerase chain reaction. After checking the size and absence of primer dimers with Bioanalyzer 2100 DNA High Sensitivity chip (Agilent Technologies, Santa Clara, CA, USA), the libraries were quantified by qPCR using Kapa Library Quantification Illumina/ABI Prism Kit protocol (KAPA Biosystems, Wilmington, MA, USA). Qualified libraries were then pooled in equimolar quantities and loaded with 15% Illumina PhiX control DNA library on a NovaSeq6000 instrument (Illumina, San Diego, CA, USA) to generate paired-end reads (150 bp). Library preparation and whole genome bisulfate sequencing (WGBS) were performed by The Centre for Applied Genomics, The Hospital for Sick Children, Toronto, Canada (http://www.tcag.ca/, accessed on 22 November 2020).

### 2.4. Identification of Methyl-Cytosine

FastQC v0.11.9 [23] was used to generate initial quality report of the raw WGBS reads, followed by trimming of adapters and low-quality sequences (reads with Phred quality score less than 30 and reads with N content greater than 8% removed) with Trim Galore (v0.6.4_dev) [24]. The cleaned pair reads of each sample were merged and mapped to the bovine reference genome (ARS-UCD1.2) using BWA v0.7.17-r1188 [25] and Samtools v1.9 under Bismark (v0.22.3). Duplicate reads were removed with Picard MarkDuplicates (v1.5) program. Methylation information was extracted with Bismark methylation extractor. The analysis workflow was coordinated with the nf-core/methylseq pipeline (v1.5) [26]. In order to decrease the possible impact of severe bias towards non-methylation at the end of reads caused by end repairing, the first 2 bp at the paired-end reads were removed.

### 2.5. Global Comparison of Methylated Sites between Different Groups

The methylation sites with coverage depth ≥ 10 × among all samples were used for global comparison. Detection of differentially methylated sites (DMCs) was accomplished with the R package, methykit v 3.12, based on three comparisons: HMF vs. LMF, HMP vs. LMP, and HMFP vs. LMFP. Parity and days in milk (DIM) were included as factors for batch effect control during analysis. Significant DMCs were defined as having a *q* value < 0.1 after Bonferroni correction for false discovery rate [27].

Hypermethylation and hypomethylation were defined as DMCs having more than 20% difference in methylation levels between groups. The genome structure annotation files containing information about genes and genomic elements, including promoter, exon, intron, 3′UTR, 5′UTR, downstream and intergenic regions were downloaded from the NCBI database (https://www.ncbi.nlm.nih.gov/genome/?term = ARS-UCD1.2, accessed on 22 November 2020) [28]. Promoters were defined as the two kb region upstream of the transcription start site (TSS) of genes while the downstream was defined as the two kb region downstream of the transcription termination site (TTS). 

### 2.6. DMCs and Quantitative Trait Loci (QTL) Co-Location Analysis

DMCs identified as significant were used for co-localization analysis with QTLs related to bovine milk production. QTL data were downloaded from the cattle QTLdb (https://www.animalgenome.org/cgi-bin/QTLdb/BT/index, accessed on 11 December 2020).

### 2.7. Statistical Overrepresentation Analysis for DMC Genes 

Genes harboring DMCs, here referred to as DMC genes were submitted for statistical overrepresentation in gene ontology (GO) terms and Reactome pathways using PANTHER (Protein Analysis Through Evolutionary Relationships, http://pantherdb.org/, accessed on 3 March 2021) [29]. The *p* values from the statistical overrepresentation tests were adjusted by Bonferroni correction for false discovery rate [27]. GO terms, including biological process (BP), molecular function (MF) and cellular component (CC), and Reactome pathways were considered to have a significant over- and underrepresentation at adjusted *p* value < 0.05.

## 3. Results 

### 3.1. Genome-Wide DNA Methylation Landscape of Mammary Gland Tissue

The genome-wide DNA methylation analysis by WGBS generated about 192,255,638 clean reads per sample with aligned rate ranging from 48.98% to 53.61% (Table S1). Only the uniquely aligned reads (92.84% of total aligned reads on average) were kept for the identification of methylated sites, including CpG (cytosine-phosphate-guanosine or 5′-C-phosphate-G-3′), CHG (5′-C-phosphate-H-G-3′, H represents A, T, or C [adenine, thymine or cytosine, respectively]) and CHH (5′-C-phosphate-H-H-3′) (Figure S1, Table S1). The number of identified cytosine (C) sites in the CpG sense were the least (4.76% of all identified C) compared with cytosine sites in the CHG (21.93%) or CHH (73.31%) sense (Figure S1). However, methylated sites were highest in the CpG sense (71.81%) compared with 1.3% and 2.18% in the CHG and CHH sense, respectively.

**Figure 1:**
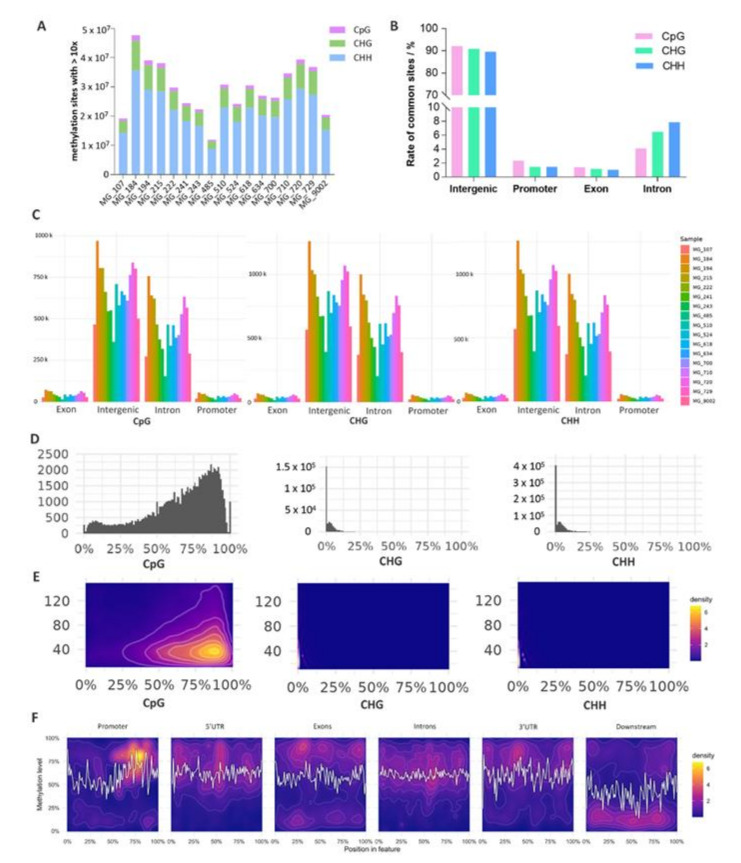
The genome-wide DNA methylation profiles of mammary gland tissues: (**A**) the number of methylated sites with ≥10 × coverage for each sample. (**B**) Distribution of methylated sites identified in all the samples in genic regions, including intergenic, promoter, intron and exon regions. (**C**) Distribution of methylated sites with ≥ 10 × coverage in genic regions for each sample. (**D**) Methylation level distribution of CpG, CHG and CHH sites; ordinate represents the count of methylated sites while abscissa represents the methylation level (percentage). (**E**) Density distribution of methylated sites. The ordinate represents coverage depth, the background color represents the normalized density while the abscissa represents the methylation level. D and E are based on one randomly selected sample (MG_729) as an example. The other samples showed similar patterns and are shown in Figures S2 and S3. (**F**) The fluctuating trends of methylation levels in genic regions, including the promoter (2 kb), 5′UTR, exon, intron, 3′UTR and downstream (2 kb) regions. The fluctuating line represents the methylation level (ordinate) of relative position in the corresponding genic regions (abscissa), and the background color represents the normalized density.

Methylated sites with at least 10× coverage were selected for the construction of genome-wide DNA methylation landscape, such as distribution in genic features and methylation levels (Figure 1A, Table S2a). CpG, CHG, and CHH methylated sites showed similar tendencies with higher abundance in intergenic (~55%) and intronic (~39%) regions (Figure 1C, Table S2b–d). Higher methylation levels were recorded for CpG sites than CHG and CHH sites (Figure S2). The number of methylated sites identified in genic regions was highly diverse between samples (Figure 1C). The methylation sites identified in all the samples were 87,096 CpG, 284,900 CHG and 818,760 CHH sites. Among them, approximately 90% of CpG (92.11%), CHG (90.88%) and CHH (89.61%) sites were in the intergenic regions (Figure 1B). Considering genic regions, CpG sites were more abundant in the promoter regions (2.35%) than CHG (1.46%) and CHH (1.47%) sites, meanwhile CHG (6.49%) and CHH (7.86%) sites were more abundant in introns than CpG (4.11%) sites (Figure 1B, Table S2e). Moreover, CpG sites had higher methylation levels and densities, as compared to CHG and CHH sites with low methylation levels and densities (Figure 1D,E, Figures S2 and S3, Table S2f). The 5‘UTR region had the highest CpG methylation level (62.33 ± 12.44%), followed by the 3′UTR and introns, while the promoter and downstream regions had relatively lower CpG methylation levels (Table S2f). Furthermore, the methylation level of CpG sites in introns and exons were relatively stable (59.32 ± 10.40%), while CpG methylation levels in the promoter (51.28 ± 20.36%) and downstream regions (47.43 ± 20.46%) fluctuated slightly around 50% (Figure 1F, Table S2f).

### 3.2. Differential Methylation between High and Low Milk Fat Producing Cows

**Figure 2:**
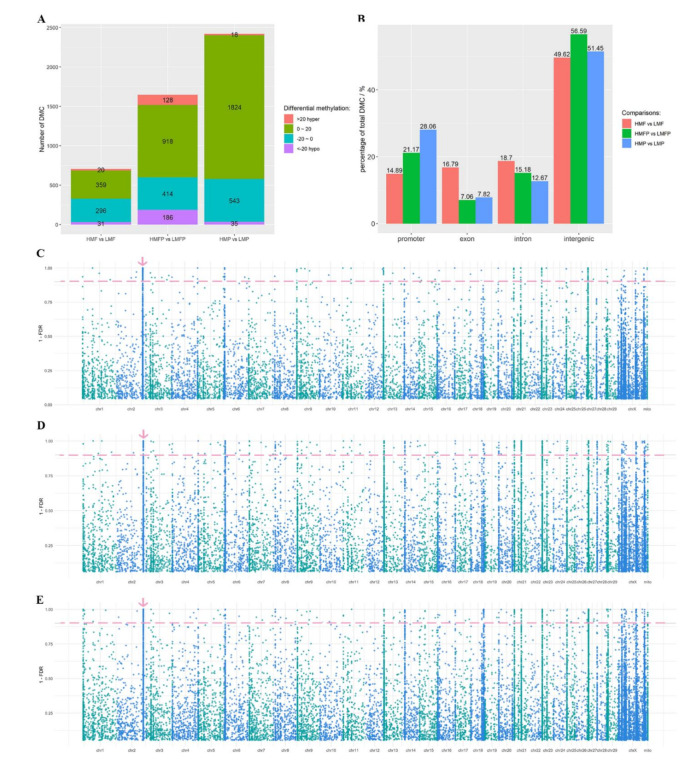
Differentially methylated CpG sites (DMC) in studied tissues. (**A**) General count of differentially methylated cytosines (DMCs) identified between mammary gland tissues producing milk with high and low fat contents (HMF vs. LMF), high or low milk protein and fat contents (HMFP vs. LMFP), as well as high and low milk protein contents (HMP vs. LMP). DMCs whose methylation levels were higher or increased by ≥ 20% in the high milk fat and/or protein groups than the low milk fat and/or protein groups were defined as hypermethylated sites. While DMCs having lower or decreased methylation levels of ≥ 20% in the high milk fat and/or protein groups were defined as hypomethylated sites. (**B**) The distribution of DMCs identified from the three comparisons in genic regions. (**C–E**) Manhattan plots showing genome-wide distribution of DMCs identified from HMF vs. LMF, HMP vs. LMP and HMFP vs. LMFP, respectively. Dots above the pink line represent DMCs at *q* value < 0.1. Many DMCs were clustered on several regions of chromosomes with similar distribution patterns in the three comparisons. The pink arrow shows one example of a DMC cluster on chromosome 2.

The comparison of methylation levels between methylated sites in mammary gland tissues of HMF and LMF group revealed 706 significant DMCs (*q* value < 0.1), including 379 and 327 DMCs having higher and lower methylation levels in HMF group compared with LMF group, respectively (Table S3a, Figure 2A). The annotation of DMCs to genic regions revealed that majority of DMCs (about 50%) were in intergenic regions, followed by promoter, intron and exon regions (Figure 2B). The DMCs were not only distributed in chromosomes (Chr) but also in unplaced genomic scaffolds. Chr2 had the most DMCs (*N* = 116), followed by Leftover_ScbfJmS_792 (*N* = 37) and Chr27 (*N* = 36) (Table S4a). On most Chr (e.g., Chr 2, 6, 9, 13, 14, 18, 20, 21, 23, 27, 29, X and mitochondria), many DMCs were concentrated in the same region. The clusters were similarly distributed amongst the three comparisons (Figure 2C–E). A total of 27 DMC genes harboring DMCs in their promoters and gene bodies, including four DMC genes harboring ≥ 3 DMCs are listed in Table 2 and Table S5a. LOC112442278, a basic proline-rich protein-like located in Chr2, harbored the most DMCs (*N* = 112) including 96 DMCs with increased methylation levels in HMF group. MIR2887-1 located downstream to LOC112442278 harbored four DMCs (Table 2, Figure S4). Following LOC112444653, a 5.8S ribosomal RNA gene located in Chr27 harbored 13 DMCs including 12 DMCs with increased methylation levels in mammary gland tissues of HMF group compared to LMF group (Table 2). In addition, phosphodiesterase 5A (PDE5A) harbored six DMCs. Overall, more hypomethylated DMC sites (31) were found compared to hypermethylated DMC sites (20) considering sites with more than 20% difference between HMF and LMF (Table S6a).

### 3.3. Differential Methylation between High and Low Milk Protein Producing Cows

**Figure 3:**
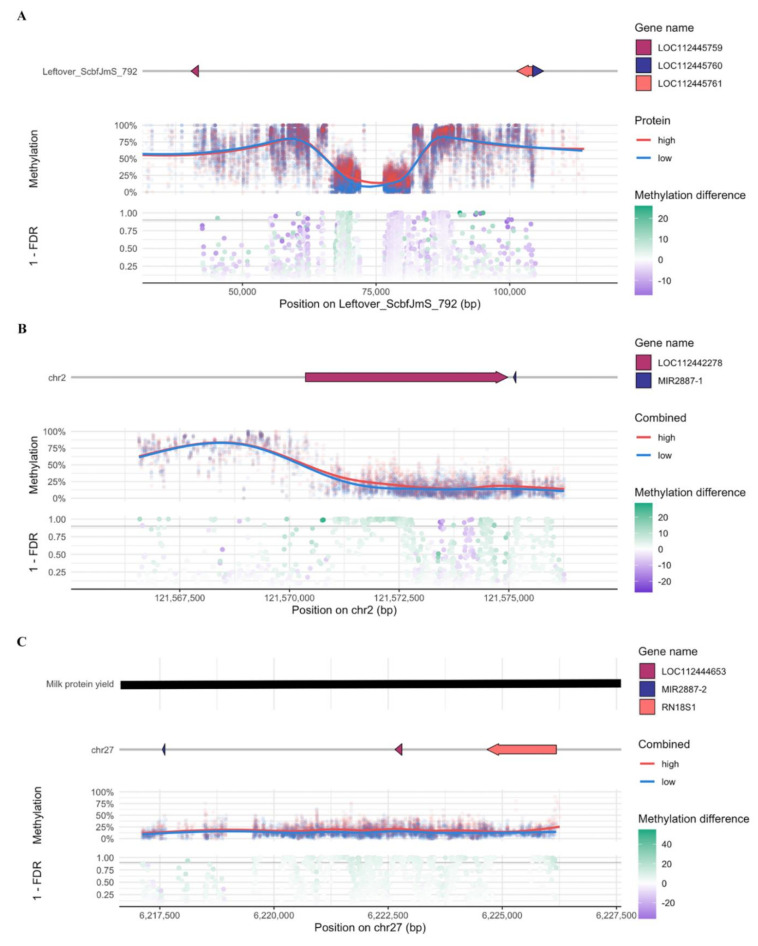
The DNA methylation states of selected regions. (**A**) CpG methylation comparison between mammary gland tissues producing milk with high and low protein contents (HMP vs. LMP) in Leftover_ScbfJmS_972 (50–100 kb). (**B**) methylation comparison between HMFP vs. LMFP in Chromosome 2 (12,1570–12,1575 kb). In A and B, the upper part shows the position of genes, and the lower part shows the methylation status of DMCs, including the methylation level and the methylation difference between the two groups. (**C**) A view of chromosome 27 (6217–6227 kb) showing the location of a QTL related to milk protein yield (upper), genes (middle) and the methylation status of DMCs identified from HMP vs. LMP comparison (lower).

In total, 2420 DMCs were identified between mammary gland tissues of cows producing milk with high and low protein contents (*q* value < 0.1) (Table S3b). There were 1842 DMCs with increased methylation levels and 578 DMCs with decreased methylation levels in HMP group compared with LMP group (Table S3b) (Figure 2A). The most DMCs were annotated to intergenic regions (51.54%) followed by the promoter regions of genes (28.06%) (Figure 2B). Moreover, more DMCs were located in Chr27 (*N* = 535), followed by Leftover_ScbfJmS_792 (*N* = 371) and Chr2 (*N* = 347) (Table S4b, Figure 2D). DMCs in a region of Leftover_ScbfJmS_792 with generally low methylation levels is located between three new uncharacterized genes (LOC112445759, LOC112445760 and LOC112445761) (Figure 3A). Fifty-six genes, including 23 novel genes, harbored at least one DMC (Table S5b). Seven DMC genes (each harbored three or more DMCs) between HMP and LMP producing cows are listed in Table 2. LOC112442278 harbored the most DMCs (*N* = 328) followed by LOC112444653 (211 DMCs), RN18S1 (60 DMCs) and two miRNAs- MIR2887-1 with 84 DMCs and MIR2887-2 with14 DMCs; and all having higher methylation levels in HMP group. Overall, more hypomethylated DMC sites (35) were found compared to hypermethylated DMCs (18) considering sites with more than 20% difference between HMP and LMP (Table S6b).

### 3.4. Differential Methylation between Cows Producing Milk with High Fat/Protein and Low Fat/Protein Contents

A total of 1645 CpG sites were differentially methylated between HMFP and LMFP groups (*q* value < 0.1) (Figure 2A, Table S3c). Majority of DMCs (56.59%) were annotated to intergenic regions, followed by promoter regions (21.17%) and introns (15.18%) (Figure 2B). Most of the DMCs were distributed on Chr27, Chr2 and Leftover_ScbfJmS_792, containing 302, 191 and 143 DMCs, respectively (Figure 2E, Table S4c). Sixty-seven genes, including 26 novel genes, were identified with at least one DMC (Table S5c). LOC112442278, LOC112444653, RN18S1 and MIR2887-1 had more DMCs than other genes. LOC112442278 harbored the most DMCs, including 168 DMCs with increased methylation levels and six DMCs with decreased methylation levels in HMFP group compared with LMFP group (Table 2). MIR2887-1, located in the downstream region of LOC112442278 (Figure 3B), harbored 30 DMCs and had increased methylation levels. LOC112444653, a 5.8S ribosomal RNA identified in Chr27, harbored 108 DMCs and with higher methylation levels in HMFP group compared with LMFP group. MIR2887-2 located at the downstream of LOC112444653, harbored five DMCs with high methylation levels (Figure 3C). In addition, RN18S1, a ribosome RNA gene, located in the upstream of LOC112444653 harbored 35 DMCs with higher methylation level in HMFP group. Overall, there were also more hypomethylated DMC sites (186) compared to hypermethylated DMCs sites (122) considering sites with more than 20% difference between HMFP and LMFP (Table S6c).

### 3.5. Overrepresented Gene Ontology Terms and Pathways by DMC Genes

The statistical overrepresentation test results of the three comparisons are listed in Table 3. For HMF vs. LMF, no GO term was significantly overrepresented by DMC genes and no reactome pathway was significantly over- or underrepresented by DMC genes from all three comparisons. Five GO-BP terms were significantly overrepresented by DMC genes from HMP vs. LMP, including four with aerobic electron transport related functions (aerobic electron transport chain (GO:0019646, adjusted *p* value = 0.0355), aerobic respiration (GO:0009060, adjusted *p* value = 0.0335), oxidative phosphorylation (GO:0006119, adjusted *p* value = 0.0085), and ATP (adenosine triphosphate) metabolic process (GO:0046034, adjusted *p* value = 0.0068)). Another significantly overrepresented GO-BP term was mitochondrial ATP synthesis coupled electron transport (GO:0042775, adjusted *p* value = 0.0355). In addition, electron transfer activity (GO:0009055, adjusted *p* value = 0.0046) and respirasome (GO:0070469, adjusted *p* value = 0.0062) were the only significantly overrepresented GO-MF and GO-CC terms, respectively. 

Besides, a total of eight GO-BP, one GO-MF and five GO-CC terms were overrepresented by DMC genes from HMFP vs. LMFP. All nine GO-BP terms were related to ATP synthesis, transport and metabolic process, such as the most significantly overrepresented oxidative phosphorylation (GO:0006119, adjusted *p* value = 0.0007) and energy derivation by oxidation of organic compounds (GO:0015980, adjusted *p* value = 0.0010) terms. Same as with HMP vs. LMP, electron transfer activity (GO:0009055, adjusted *p* value = 0.0004) was the only significantly overrepresented GO-MF term for HMFP vs. LMFP. The top three significantly overrepresented GO-CC terms were related to respirasome, including respirasome (GO:0070469, adjusted *p* value = 0.0000), respiratory chain complex (GO:0098803, adjusted *p* value = 0.0003) and mitochondrial respirasome (GO:0005746, adjusted *p* value = 0.0066).

### 3.6. Overlapping or Co-Located DMCs with QTLs Related to Milk Production Traits

**Figure 4:**
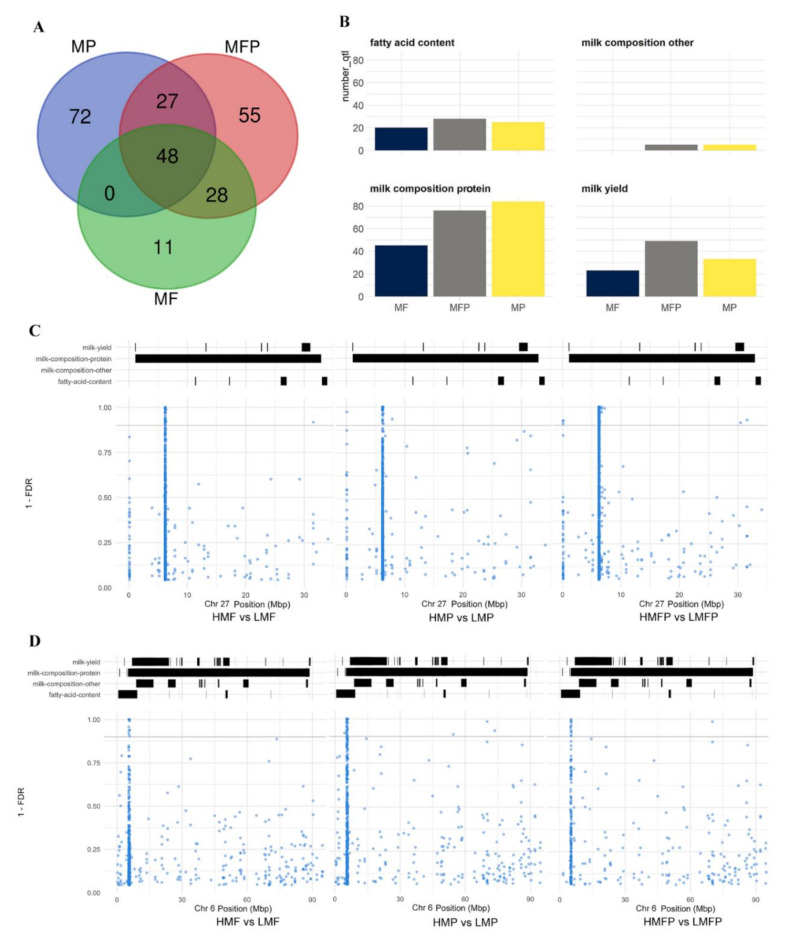
QTLs overlapped or co-located with DMCs. (**A**) Venn diagram showing the number of QTLs uniquely and commonly overlapped with DMCs identified from HMF vs. LMF, HMP vs. LMP and HMFP vs. LMFP comparisons. MP: milk protein, MF: milk fat, MFP: milk fat and protein. (**B**) The number of QTLs co-located with DMCs from HMF vs. LMF, HMP vs. LMP and HMFP vs. LMFP comparisons. (**C**) A view of a region of chromosome 27 with a cluster of DMCs identified from the three comparisons. (**D**) A view of a region of chromosome 6 with a cluster of DMCs identified from the three comparisons. The upper parts of C and D show QTL locations catalogued in the animal genome database (www.animalgenome.org, accessed on 11 December 2020) and filtered to include only loci marked as “significant” and the lower parts are annotated CpG sites (blue dots).

A total of 242 milk production related QTLs were found to overlap with at least one DMC. Specifically, 87, 147 and 158 QTLs overlapped with DMCs from HMF vs. LMF (*N* = 83), HMP vs. LMP (*N* = 708) and HMFP vs. LMFP (*N* = 408), respectively, comparisons (Figure 4A, Table S7). Approximately half of the co-located QTLs (*N* = 122) are related to milk-composition-protein, followed by milk yield (*N* = 68), fatty acid content (*N* = 44) and milk-composition-other (*N* = 8) (Figure 4B). Forty-nine QTLs overlapped with DMCs from all three comparisons, and 10 QTLs overlapped with more than three DMCs from each comparison (three QTLs in Chr27, six QTLs in Chr6 and one QTL in Chr3) (Table 4). Moreover, a cluster of DMCs in Chr27 overlapped with QTLs related to milk-composition-protein (Figure 4C). The three QTLs on Chr27 (QTL-2611, QTL-10104 and QTL-2592) were co-located with a higher number of DMC sites than other QTLs (Table 4). For example, QTL-10104, a QTL for milk protein yield in Chr27, overlapped with the most DMCs, including 36 from HMF vs. LMF, 534 from HMP vs. LMP and 299 from HMFP vs. LMFP. Following, QTL-2611 (milk protein yield) and QTL-2592 (milk yield), located at the same position in Chr27 and also overlapped with QTL-10104, are co-located with 35 DMCs from HMF vs. LMF, 533 DMCs from HMP vs. LMP and 297 DMCs from HMFP vs. LMFP. In addition, three QTLS, QTL-12232 (stearic aldehyde content), QTL-12233 (docosahexaenoic acid content) and QTL-12234 (omega-6 to omega-3 fatty acid ratio) located at the same position in Chr6 overlapped with 11 DMCs from HMF vs. LMF, 11 DMCs from HMP vs. LMP and 10 DMCs from HMFP vs. LMFP (Figure 4D).

To further understand the potential functions of DMC genes, we identified DMC genes that were overlapped or co-located with QTLs. A total of 7, 20 and 17 genes were identified that harbored DMCs and also located in the same regions as QTLs from HMF vs. LMF, HMP vs. LMP and HMFP vs. LMFP, respectively (Table S8). Six DMC genes harbored ≥ 3 DMCs from at least one comparison (Table 5), including two ribosomal RNA genes (RN18S1 and LOC112444653), phosphodiesterase 5A (PDE5S), F-box protein 16 (FBXO16), defensin β 7 (DEFB7) and one uncharacterized gene (LOC101907803). RN18S1 harbored one DMC located at position 6224783 of Chr27, whose methylation level was significantly different in all three comparisons. It also harbored additional 59 DMCs from HMP vs. LMP and 34 DMCs from HMFP vs. LMFP that were also differentially methylated when comparing HMP with LMP. Similarly, LOC112444653 harbored one DMC (Chr27:622688) that was differently methylated in all three comparisons, and another 15 DMCs that were common to the comparisons HMP vs. LMP and HMFP vs. LMFP. Furthermore, DMCs in RN18S1 and LOC112444653 overlapped with QTL-10104 for milk protein yield (Figure 3C). Besides, PDE5A harbored six DMCs (HMF vs. LMF), four DMCs (HMP vs. LMP) and five DMCs (HMFP vs. LMFP) with one DMC (Chr6:5790277) common to all three comparisons. These DMCs were overlapped with QTLs for fatty-acid-content (QTL-12232, QTL-12233 and QTL-12234) and milk-composition-protein (QTL-10148 for milk protein yield and QTL-10208 for milk protein percentage).

## 4. Discussion

During the last century, milk yield was the focus point of genetic selection in dairy cattle which today has led to doubling of the average milk produced by a Holstein cow compared to the 1950s [7,30,31]. Recently, milk composition, especially protein and fat contents, is attracting increased attention due to consumer demands for healthier milk products. With the knowledge that epigenetic factors are important regulatory mechanisms of mammary gland productivity (reviewed by [15]), quantifying the associated epigenetic variations will provide additional information underlying phenotype diversity and will complement genomic information to drive-up improve gain in genetic selection for enhanced milk components [8,31]. In this study, we focused on quantifying the DNA methylation alterations in mammary gland tissues where milk components are synthesized and/or collated and secreted. Understanding the DNA methylation variations across tissues, which is crucial for cell differentiation, is important for investigating the potential regulatory roles of epigenetic factors in complex traits, such as diseases and milk production [15,32]. Therefore, compared with other tissues, for example peripheral blood, quantifying DNA methylation marks in mammary gland is more direct and convincing to understand its roles in milk production. It should be noted that the mammary gland is a complex organ and that total DNA isolated from this complex tissue was used in this study therefore, it is possible that the DNA methylation patterns could be coming from different cell types. More research is therefore needed to characterize the DNA methylation patterns of the diverse cell populations constituting mammary gland tissue and their roles in milk fat and/or protein production. Such an approach will provide further in-depth and comprehensive understanding of DNA methylation roles in milk fat and/or protein production.

The cattle methylome has been reported in different tissues, such as muscle, mammary gland, and peripheral blood, revealing consistent features that are shared with other mammals [32,33,34,35]. The methylation landscape of mammary gland tissue in this study also found some consistent features. For example, more methylated sites, including CpG, CHG and CHH, were identified in introns than exons, which may be due to the longer lengths of introns than exons, as previously noted by Dechow and Liu [17]. The downstream region had lower methylation level, which is similar to observations in human, rat and sheep [36,37,38]. In addition, lower methylation level and higher density of methylation sites in the promoter region than in exons and introns observed in this study (Figure 1F), confirms observations in other species [33,39]. This is because more than two-thirds of promoter regions contain CpG islands, which are rich in CpG sites, but which are also rarely methylated [40]. DNA methylation in promoter regions has shown important interactions with transcriptional activities, such as the inhibition of the binding of transcription factors, and thereby affect gene expression in response to internal or external environmental changes [41,42].

The genome-wide DNA methylation patterns of mammary glands producing milk with various fat/protein contents were compared to identify DMCs that may influence the synthesis of milk protein or fat. Approximately half of the DMCs out of 706, 2420, and 1645 DMCs identified from the comparisons of HMF vs. LMF, HMP vs. LMP, and HMFP vs. LMFP, respectively, are located in intergenic regions, while only about 20% are located in promoter regions, thereby corroborating previous reports in dairy and goat mammary gland tissues [36,43]. This report also corroborates data on single nucleotide polymorphism (SNP) mapping in cattle and humans [44,45,46]. The HMF vs. LMF comparison revealed the least DNA methylation difference compared with HMP vs. LMP, and HMFP vs. LMFP comparisons while HMP vs. LMP comparison revealed the most DMCs. DMC genes from HMP vs. LMP comparison were significantly overrepresented in GO terms for electron transfer activity, ATP synthesis and metabolic process. Similarly, DMC genes from HMFP vs. LMFP comparison showed significant overrepresentation of GO terms for ATP synthesis coupled electron transport, while no GO terms were enriched by genes from HMF vs. LMF. The energy level in cow’s diet has been found to affect the yield of protein in milk [47,48]. During lactation, the protein and energy needs of cows are increased about five times to support increased mRNA translation activities and milk protein synthesis [49]. Our data suggest that DNA methylation may affect milk protein production by regulating energy availability, such as biological processes related to ATP synthesis and metabolism. In addition to the increased energy requirements for protein synthesis during milk production, the process of milk protein and fat synthesis may have possible competition for energy in the mammary gland [50]. The mitochondrial functions include ATP production, metal homeostasis, regulation of cellular metabolism, and cellular respiration. The main modifications that regulate gene expression (gene activation or silencing) within the mitochondria include DNA methylation (5 mC and 6 mA methylation, where 5 mC is 5-methyl cytosine and 6 mA is 6-methyl adenine), noncoding RNAs, and post-translational modifications of nucleoid proteins [51]. Because mitochondria are key organelles in the cell that carry out many important functions necessary for cell survival, and given its important role of energy supply regulation in the mammary gland, it is thus not surprising that epigenetic modifications of mitochondrial functions were affected in this study.

DMC genes with the most DMCs from the three comparisons were all ribosome RNA genes (rRNA), such as LOC112444653 and RN18S1 (Table 2). The DNA methylation alterations in rRNAs is less well-studied, and its potential roles in milk production is still unclear. However, studies in human and other model animals revealed some potential roles of DNA methylation in rRNAs, such as in silencing of the nucleolar chromatin and suppression of rRNA gene expression, amongst others [52]. For example, it was found that lack of suppression maintained by CpG methylation promotes cryptic RNA polymerase II transcription and disruption of rRNA processing, which partially explains the negative effects of losing CpG methylation frequently found in most disease conditions [53,54,55]. The DMCs in the rRNA genes had increased methylation levels, including LOC112444653 with 12, 211 and 108 DMCs from the HMF vs. LMF, HMP vs. LMP and HMFP vs. LMFP comparisons, respectively, and having increased methylation levels in the HMF, HMP and HMFP groups. Hypermethylation in rRNA has been correlated with aging and Alzheimer’s disease, suggesting the potential involvement of rRNA methylation in the regulation of gene expression and the various phenotypes that occur during a lifetime [56,57,58]. Besides, rRNA is the most abundant mRNA transcript in cells, whose transcriptional processing is crucial for keeping energy homeostasis and which could be affected by diverse environmental factors, such as nutrition and stress factors [59]. Because ribosome is a key component in protein synthesis, the altered expression of rRNA may influence the translation of mRNAs into proteins and consequently the content of milk proteins. Therefore, the DNA methylation alterations of rRNA genes could be a possible layer of regulation of rRNA synthesis, and in turn, altered ribosomal biogenesis and protein synthesis during milk production.

MIR2287 also harbored a high number of DMCs (Table 2). MIR2287 has been previously reported in bovine skeletal muscle satellite cells [60], but its potential regulatory roles are currently unknown. MiRNA, another epigenetic factor that regulates gene expression, may interact with DNA methylation in its functions [61]. DNA methylation in miRNA can inhibit the expression of miRNAs, while miRNAs can directly target DNA methyltransferases causing inhibition of DNA methylation and influencing global methylation pattern [62]. The interaction between DNA methylation and miRNA has been observed in mammary glands of dairy cows with the potential involvement in mammary gland development and lactation [63]. Therefore, the DNA methylation alterations in MIR2287 identified in this study suggest its possible effects on regulating the expression of its target genes, and possibly milk fat/protein synthesis and secretion. Immediately upstream of miR-2287-1 is LOC112442278, a basic proline-rich protein-like, that also harbored 96, 328 and 168 DMCs with increased methylation levels in HMF, HMP and HMFP groups respectively. Similarly, MIR2287-2 is located upstream of two rRNAs (LOC112444653 and RN18S1), and the methylation levels of their DMCs were all increased in mammary glands producing higher contents of milk fat/protein, suggesting their potential roles in milk fat and protein synthesis. Associations between DNA methylation alterations and genetic mutations, such as SNPs, have been observed in diverse tissues of mammals [64,65]. Moreover, the overlap between altered CpG sites and QTLs generated using SNP data suggest one possible mechanism that genetic polymorphism impacts gene expression by the altered epigenetic patterns [66,67], thereby supporting potential regulatory roles of epigenetic alterations in milk fat/protein production. 

Some DMCs reported in this study clustered in the same genomic region and chromosome (Figure 2C–E) in the same manner observed with SNP data in genome wide association studies for milk production traits [46,68,69] suggesting potential interaction between SNP and DNA methylation to influence milk production traits in cattle. Moreover, DMC and QTL co-location analysis placed some identified DMCs in the same genomic regions with reported QTLs for several milk production traits (Figure 4C,D, Table 5). Eleven DMCs in Chr6 from HMF vs. LMF comparison were co-located with three QTLs (QTL-12232, QTL-12233 and QTL-12234) for fatty acid content, suggesting the possible involvement of these DMCs in regulating milk fat synthesis. Ten QTLs for milk protein were found to harbor more than 10 DMCs from HMP vs. LMP comparison, represented by QTL-10104 and QTL-2611 co-located with 534 and 533 DMCs, respectively. This suggests that DNA methylation may play regulatory roles during milk protein production. Noteworthy is QTL-10104 for milk protein that is located on a region (1.1–32.9 Mbp) of chromosome 27 with the most DMCs from all three comparisons, indicating possible interactions between genetic polymorphisms and DNA methylation alterations during milk fat and/or protein production. QTL-2611 (milk protein yield) and QTL-2592 (milk yield) are also located within this region of chromosome 27, suggesting the potential involvement of DMCs in this region in the regulation of milk protein content and milk yield. Moreover, the rRNA genes (RN18S1 and LOC112444653) harboring the most DMCs overlapped with these three QTLs, further supporting the notion that, potential interaction between DNA methylation changes, rRNA expression and SNPs (QTLs) may be a possible regulatory mechanism underlying milk protein production. Another example worthy of attention is PDE5A, previously reported as involved in the regulation of milk production [70], harbored more than three DMCs from each of the three comparisons and overlapped with six QTLs in this study. The QTLs included three QTLs for milk protein (QTL-10148, QTL-10208 and QTL-6065) and another three for milk fatty acid content, such as stearic aldehyde content (QTL-12232), docosahexaenoic acid content (QTL-12233), and omega-6 to omega-3 fatty acid ratio (QTL-12234). This reveals the possibility that, DNA methylation alterations in this gene may interact with SNPs (QTLs) to affect milk fat and protein production. Our data is supported by reported associations between SNP and differential DNA methylation in humans [64,65], and indication that DNA methylation alteration due to SNPs cause variable expression of related genes and differential phenotype expression.

## 5. Conclusions

Differential DNA methylation patterns were detected in mammary gland tissues from Canadian Holstein cows producing milk with various fat and protein contents. Some identified DMCs were co-located with QTLs for milk production traits, including QTLs for milk protein and milk fat, suggesting potential involvement of DNA methylation alterations in the genetic variation underlying milk fat and protein yields, and mammary gland biological processes. Considering cell type differences in mammary gland tissue and the small number of samples analyzed, more studies and a higher sample size are needed for more in-depth and comprehensive understanding of DNA methylation involvement in milk fat and protein production.

## Figures and Tables

**Table 1:** Test day milk fat and protein composition (mean of four consecutive months values) of experimental animals.

Animal ID	Phenotype/Analysis Group ^1^	Parity	DIM ^2^	Fat (%) ^2^	Protein (%) ^2^
High milk fat (HMF) Group					
241	**HMF**-HMP	3	262	**5.55 ± 0.38**	4.1 ± 0.22
194	**HMF**-HMP	4	216	**5.20 ± 0.18**	3.83 ± 0.22
184	**HMF**	5	264	**5.09 ± 0.29**	3.40 ± 0.14
215	**HMF**	4	89	**5.05 ± 0.07**	2.98 ± 0.21
524	**HMF**	3	145	**4.93 ± 0.66**	3.28 ± 0.35
222	**HMF**	4	230	**4.90 ± 0.655**	3.39 ± 0.096
700	**HMF**-HMP	1	318	**4.83 ± 1.18**	3.83 ± 0.29
Low milk fat (LMF) group					
618	**LMF**-LMP	2	155	**3.40 ± 0.22**	3.15 ± 0.13
729	**LMF**-LMP	1	99	**3.55 ± 0.37**	2.93 ± 0.22
243	**LMF**-LMP	3	205	**3.64 ± 0.15**	3.11± 0.13
634	**LMF**	2	58	**3.70 ± 0.31**	3.3 ± 0.377
107	**LMF**	7	203	**3.70 ± 2.05**	3.28 ± 0.28
High milk protein (HMP)group					
241	HMF-**HMP**	3	262	5.55 ± 0.38	**4.1 ± 0.22**
194	HMF-**HMP**	4	216	5.20 ± 0.18	**3.83 ± 0.22**
700	HMF-**HMP**	1	318	4.83 ± 1.18	**3.83 ± 0.29**
9002	**HMP**	3	48	4.58 ± 0.49	**3.70 ± 0.848**
710	**HMP**	1	352	4.38 ± 0.096	**3.63 ± 0.08**
Low milk protein (LMP) group					
720	**LMP**	1	111	4.7 ± 0.65	**2.9 ± 0.32**
729	LMF-**LMP**	1	99	3.55 ± 0.37	**2.93 ± 0.22**
215	HMF-**LMP**	4	89	5.05 ± 0.07	**2.98 ± 0.21**
510	**LMP**	3	192	4.58 ± 0.63	**3.00 ± 0.19**
485	**LMP**	4	132	4.33 ± 0.32	**3.00 ± 0.43**
243	LMF-**LMP**	3	205	3.64 ± 0.15	**3.11 ± 0.13**
618	LMF-**LMP**	2	155	3.40 ± 0.22	**3.15 ± 0.13**
High milk fat and protein (HMFP) group					
241	**HMF-HMP**	3	262	**5.55 ± 0.38**	**4.1 ± 0.22**
194	**HMF-HMP**	4	216	**5.20 ± 0.18**	**3.83 ± 0.22**
700	**HMF-HMP**	1	318	**4.83 ± 1.18**	**3.83 ± 0.29**
Low milk fat and protein (LMFP) group					
618	**LMF-LMP**	2	155	**3.40 ± 0.22**	**3.15 ± 0.13**
729	**LMF-LMP**	1	99	**3.55 ± 0.37**	**2.93 ± 0.22**
243	**LMF-LMP**	3	205	**3.64 ± 0.15**	**3.11 ± 0.13**

^1^ HMP: high milk protein (>3.60%); LMP: low milk protein (≤3.15%); HMF: high milk fat > 4.80%); LMF: low milk fat (≤3.70%). Some cows belonged to more than one group. The bold font shows the group and the corresponding milk fat or protein contents on which the analysis is based. ^2^ DIM: days in milk. ^2^ fat % and protein %: mean ± SD.

**Table 2:** DMC genes with at least three differentially methylated CpG (DMC) sites.

Comparison ^1^	Gene Name	Gene Description	Chr ^2^	DMCs with Increased Methylation Level	DMCs with Decreased Methylation Level
HMF vs. LMF	LOC112442278	Basic proline-rich protein-like	chr2	96	6
	LOC112444653	5.8S ribosomal RNA	chr27	12	1
	PDE5A	Phosphodiesterase 5A	chr6	3	3
	MIR2887-1	MicroRNA mir-2887-1	chr2	1	3
HMP vs. LMP	LOC112442278	Basic proline-rich protein-like	chr2	328	0
	LOC112444653	5.8S ribosomal RNA	chr27	211	0
	MIR2887-1	MicroRNA mir-2887-1	chr2	84	0
	RN18S1	18S ribosomal RNA	chr27	60	0
	MIR2887-2	MicroRNA mir-2887-2	chr27	14	0
	LOC112443250	5S ribosomal RNA	chr21	9	1
	PDE5A	Phosphodiesterase 5A	chr6	3	1
HMFP vs. LMFP	LOC112442278	Basic proline-rich protein-like	chr2	168	6
	LOC112444653	5.8S ribosomal RNA	chr27	108	0
	RN18S1	18S ribosomal RNA	chr27	35	0
	MIR2887-1	MicroRNA mir-2887-1	chr2	30	0
	ENSBTAG00000052622.1	Novel gene (protein coding)	chr14	1	9
	PDE5A	Phosphodiesterase 5A	chr6	2	3
	MIR2887-2	MicroRNA mir-2887-2	chr27	5	0
	FBXO16	F-box protein 16	chr8	1	3
	DEFB7	Befensin β 7	chr27	0	3
	COX1	Cytochrome c oxidase subunit I	chrM	2	1
	ENSBTAG00000043565.1	Novel gene (Mt tRNA)	chrM	2	1

**^1^** HMF vs. LMF: comparison between mammary gland tissues from cows producing milk with high and low fat contents. HMP vs. LMP: comparison between mammary gland tissues from cows producing milk with high and low protein contents. HMFP vs. LMFP: comparison between mammary gland tissues from cows producing milk with both high fat/high protein and low fat/low protein contents. **^2^** chr: chromosome.

**Table 3:** Significantly overrepresented gene ontology (GO) terms by DMC genes.

Comparison ^1^	GO Identification	GO Term Name	GO Category ^2^	Fold Enrichment	+ ^3^	Adjust *p* Value
HMP vs. LMP	GO:0019646	Aerobic electron transport chain	BP	37.81	+	0.0355
	GO:0009060	Aerobic respiration	BP	20.97	+	0.0335
	GO:0006119	Oxidative phosphorylation	BP	28.02	+	0.0085
	GO:0046034	ATP metabolic process	BP	18.53	+	0.0066
	GO:0042775	Mitochondrial ATP synthesis coupled electron transport	BP	35.18	+	0.0466
	GO:0009055	Electron transfer activity	MF	25.33	+	0.0046
	GO:0070469	Respirasome	CC	20.56	+	0.0062
HMFP vs. LMFP	GO:0042773	ATP synthesis coupled electron transport	BP	34.88	+	0.0032
	GO:0006119	Oxidative phosphorylation	BP	28.15	+	0.0007
	GO:0022904	Respiratory electron transport chain	BP	26.25	+	0.0124
	GO:0009060	Aerobic respiration	BP	21.07	+	0.0036
	GO:0022900	Electron transport chain	BP	18.71	+	0.0070
	GO:0046034	ATP metabolic process	BP	18.1	+	0.0010
	GO:0045333	Cellular respiration	BP	17.58	+	0.0100
	GO:0015980	Energy derivation by oxidation of organic compounds	BP	13.65	+	0.0411
	GO:0009055	Electron transfer activity	MF	25.45	+	0.0004
	GO:0098803	Respiratory chain complex	CC	23.56	+	0.0003
	GO:0070469	Respirasome	CC	24.1	+	0.0000
	GO:0005746	Mitochondrial respirasome	CC	20.55	+	0.0066
	GO:1990204	Oxidoreductase complex	CC	17.56	+	0.0138
	GO:0098800	Inner mitochondrial membrane protein complex	CC	13.88	+	0.0415

^1^ HMF vs. LMF: comparison between mammary gland tissues from cows producing milk with high and low fat contents. HMP vs. LMP: comparison between mammary gland tissues from cows producing milk with high and low protein contents. HMFP vs. LMFP: comparison between mammary gland tissues from cows producing milk with both high fat/high protein and low fat/low protein contents. DMC: differentially methylated cytosine (CpG) sites. ^2^ BP: biological process; MF: molecular function; CC: cellular component; + ^3^: overrepresentation, i.e., more present in high vs. low.

**Table 4:** Quantitative trait loci (QTLs) co-located with more than three differentially methylated CpG sites per comparison.

QTL ID	QTL Symbol ^1^	QTL Trait Name	Chr ^2^	Start Position	End Position	QTL Trait Class	DMC (HMF vs. LMF) ^3^	DMC (HMP vs. LMP) ^4^	DMC (HMFP vs. LMFP) ^5^
10104	PY	Milk protein yield	chr27	1099081	32912109	milk-composition-protein	36	534	299
2611	PY	Milk protein yield	chr27	5484642	21801052	milk-composition-protein	35	533	297
2592	MY	Milk yield	chr27	5484642	21801052	milk-yield	35	533	297
12232	ALD-C18:0	Stearic aldehyde content	chr6	612894	9283340	fatty-acid-content	11	11	10
12233	FA-C22:6	Docosahexaenoic acid content	chr6	612894	9283340	fatty-acid-content	11	11	10
12234	N6N3R	Omega-6 to omega-3 fatty acid ratio	chr6	612894	9283340	fatty-acid-content	11	11	10
10208	PP	Milk protein percentage	chr6	5023293	18528012	milk-composition-protein	11	11	10
6065	PY	Milk protein yield	chr6	767213	13254392	milk-composition-protein	11	11	10
10148	PY	Milk protein yield	chr6	5747732	5928842	milk-composition-protein	6	4	5
2443	PP	Milk protein percentage	chr3	12993207	44877667	milk-composition-protein	3	3	4

^1^ PY: Protein yield; MY: Milk yield; ALD: Aldehyde; FA: Fatty acid; PP: Protein percentage. ^2^ chr: chromosome. **^3^** DMC: differentially methylated cytosine (CpG) site. HMF vs. LMF: comparison between mammary gland tissues from cows producing milk with high and low fat contents. ^4^ HMP vs. LMP: comparison between mammary gland tissues from cows producing milk with high and low protein contents. **^5^** HMFP vs. LMFP: comparison between mammary gland tissues from cows producing milk with both high fat/high protein and low fat/ low protein contents.

**Table 5:** Genes harboring three or more differentially methylated cytosine (CpG) sites and co-located with QTLs.

Gene ID	Gene Name	Chr ^1^	DMCs ^2^			QTL Trait Class	QTL Trait	QTL ID	Gene Function
			HMF vs. LMF	LMP vs. HMP	HMFP vs. LMFP				
RN18S1	18S ribosomal RNA	Chr27	1	60	35	Milk-composition-protein	Milk protein yield	10104; 2611	Structural constituent of ribosome
							Milk yield	2592	
LOC112444653	5.8S ribosomal RNA	Chr27	1	17	16	Milk-composition-protein	Milk protein yield	10104; 2611	Structural constituent of ribosome
							Milk yield	2592	
PDE5A	Phosphodiesterase 5A	Chr6	6	4	5	fatty-acid-content	Stearic aldehyde content	12232	Implicated in 3′,5′-cyclic-GMP phosphodiesterase activity; Involved in cGMP binding; Involved in cyclic-nucleotide phosphodiesterase activity; Involved in metal ion binding
							Docosahexaenoic acid content	12233	
							Omega-6 to omega-3 fatty acid ratio	12234	
						Milk-composition-protein	Milk protein yield	10148; 6065	
							Milk protein percentage	10208	
FBXO16	F-box protein 16	Chr8	1	1	4	Milk-yield	Milking speed	3438	Involved in protein binding
DEFB7	defensin β 7	Chr27	0	1	3	Milk-composition-protein	Milk protein yield	10104; 2611	Involved in positive chemotaxis
							Milk yield	2592	
ENSBTAG00000052622.1	Novel gene (protein coding)	Chr14	0	1	9	Milk-composition-protein; milk-yield	Milk protein percentage	2604; 3413	None
							Milk protein yield	10099, 10100, 10101	
							Milk yield	3608	

^1^ chr: chromosome. **^2^** DMC: differentially methylated cytosine (CpG) sites. HMF vs. LMF: comparison between mammary gland tissues from cows producing milk with high and low fat contents. HMP vs. LMP: comparison between mammary gland tissues from cows producing milk with high and low protein contents. HMFP vs. LMFP: comparison between mammary gland tissues from cows producing milk with both high fat/high protein and low fat/low protein contents.

## Data Availability

The datasets reported in this study have been deposited in the NCBI Sequence Read Archive (SRA) under the BioProject PRJNA749752.

## Supplementary Materials

The following are available online at https://www.mdpi.com/article/10.3390/genes12111727/s1, Figure S1: Identified methylation sites (including CpG, CHG and CHH sites) based on all the uniquely aligned reads. The inside pie represents the proportion of the three types of methylation sites (CpG, CHG and CHH) among all the identified putative methylation sites. The outer circle represents the status of methylation sites (percentage of methylated or not methylated sites) of each type of methylation site to all the identified methylation sites. Figure S2: The methylation level distribution of methylated sites with ≥ 10 × coverage according to type of methylation and sample. Figure S3: The density distribution of methylation sites according to type of methylation and sample. Figure S4: Methylation comparison between mammary gland tissues producing milk with high milk fat compared with mammary gland tissues producing milk with low milk fat content (HMF vs. LMF) in Chromosome 2 (12,1570–12,1575 kb). Table S1: Generated reads and mapping statistics of WGBS data for each sample. Table S2: Methylation sites with ≥ 10 coverage depth per samples and their annotation to genic regions. Table S3: Differentially methylated CpG sites identified from the three comparisons. Table S4: Distribution of differentially methylated sites in chromosomes and scaffolds. Table S5: Genes harboring differentially methylated CpG sites. Table S6: hyper- and hypo-methylated sites with more than 20% difference in CpG methylation. Table S7: QTLs co-located with differentially methylated CpG sites. Table S8: Genes harboring differentially methylated CpG sites and co-located with QTLs.
