# Supplementary figures and images for: Whole Genome DNA Methylation Variations in Mammary Gland Tissues from Holstein Cattle Producing Milk with Various Fat and Protein Contents
Source: Genes (Basel). 2021 Oct 28;12(11):1727. doi: 10.3390/genes12111727 (PMC8618717; doi:10.3390/genes12111727)

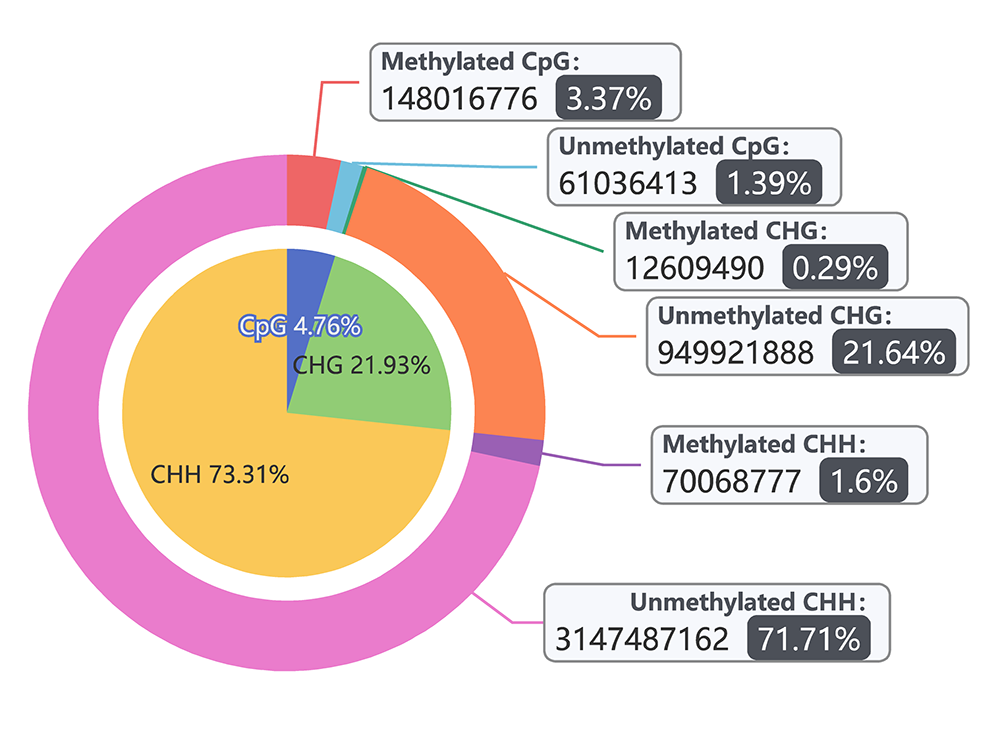

Supplement: Supplementary file 1 [file genes-12-01727-s001.zip › SupplementaryMaterial/FigureS1_methylation_identification.tif]

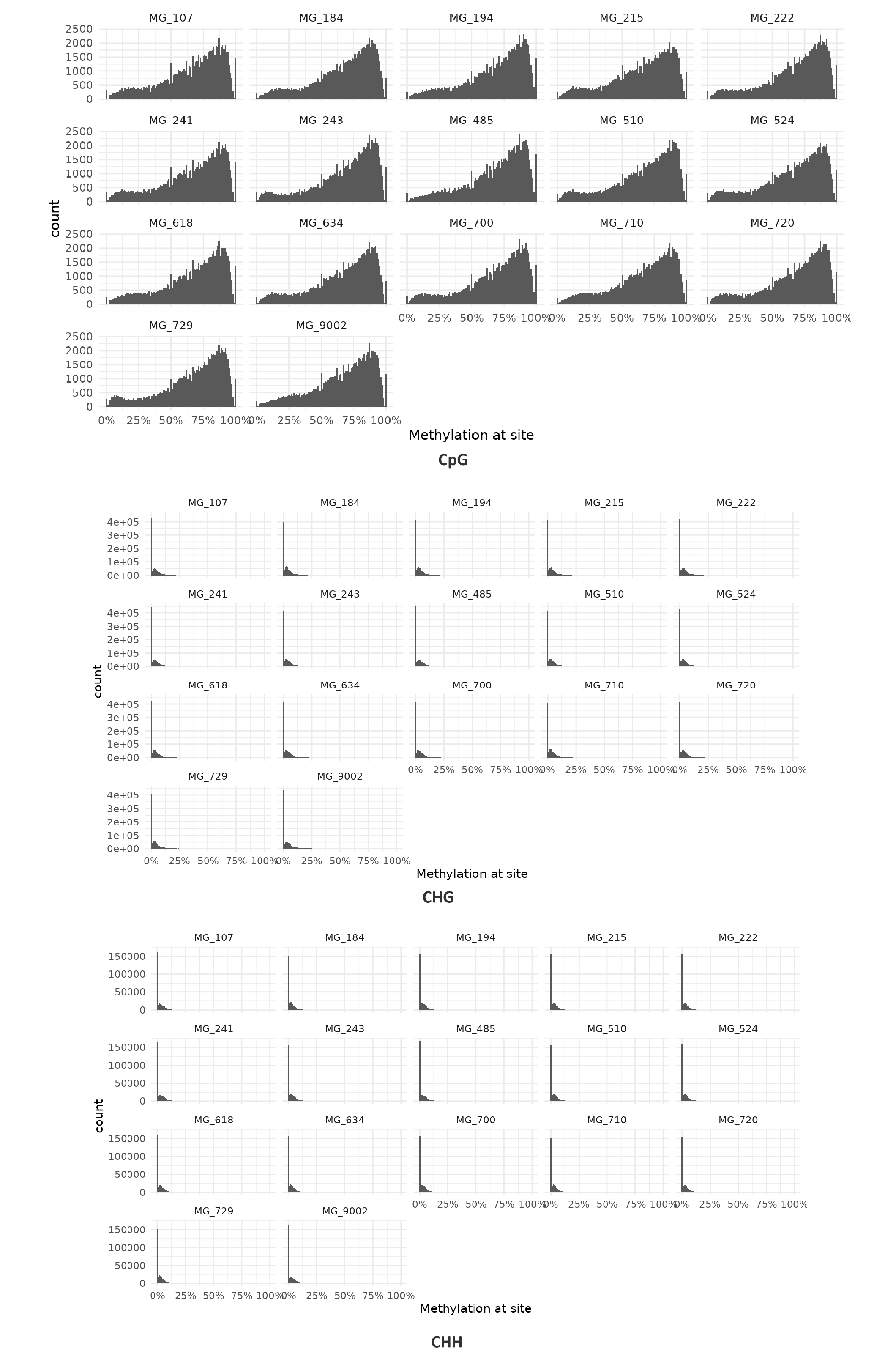

Supplement: Supplementary file 1 [file genes-12-01727-s001.zip › SupplementaryMaterial/FigureS2_methylation_level_of_common_sites.tif]

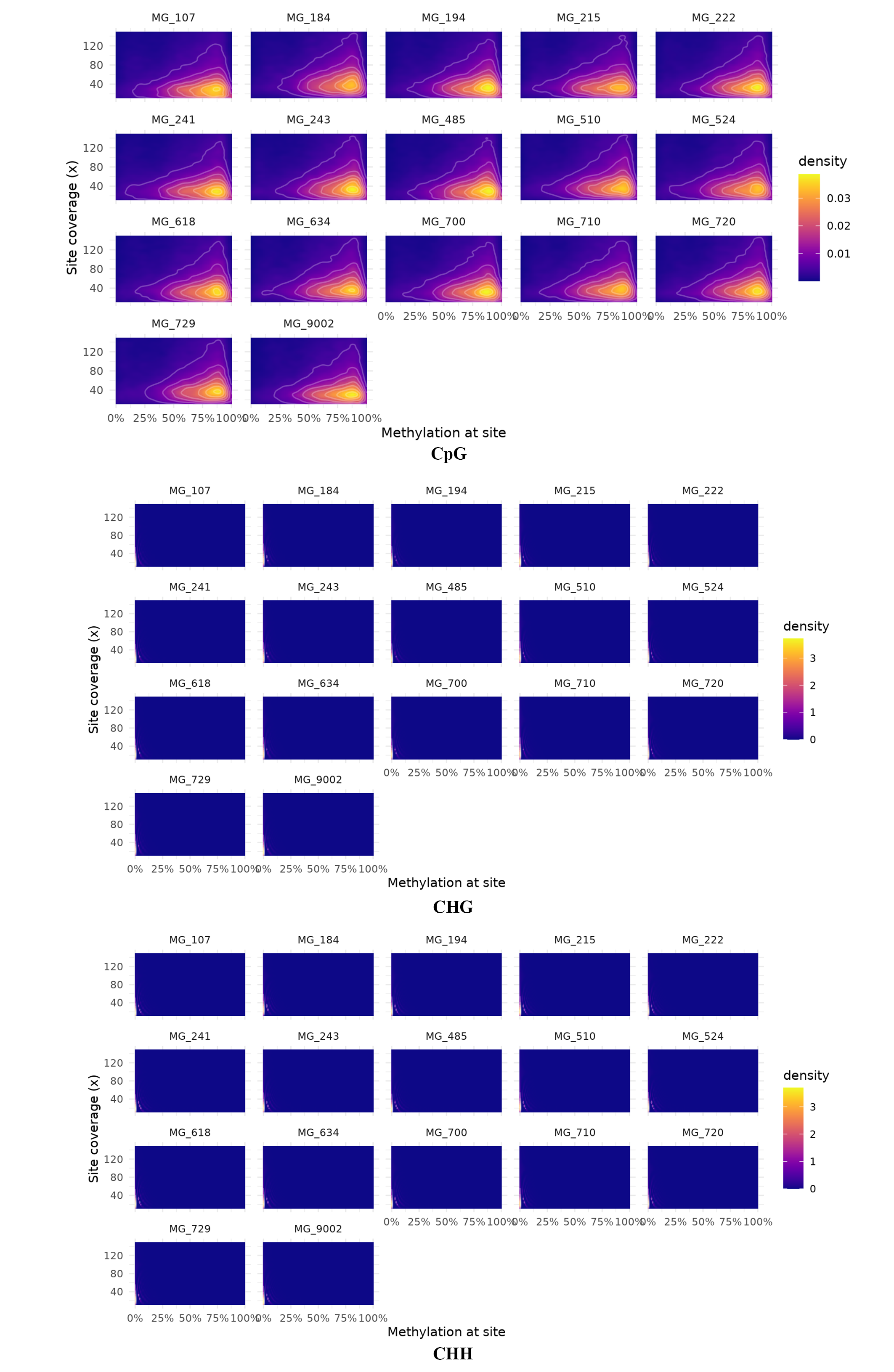

Supplement: Supplementary file 1 [file genes-12-01727-s001.zip › SupplementaryMaterial/FigureS3_methylation_density_of_common_sites.tif]

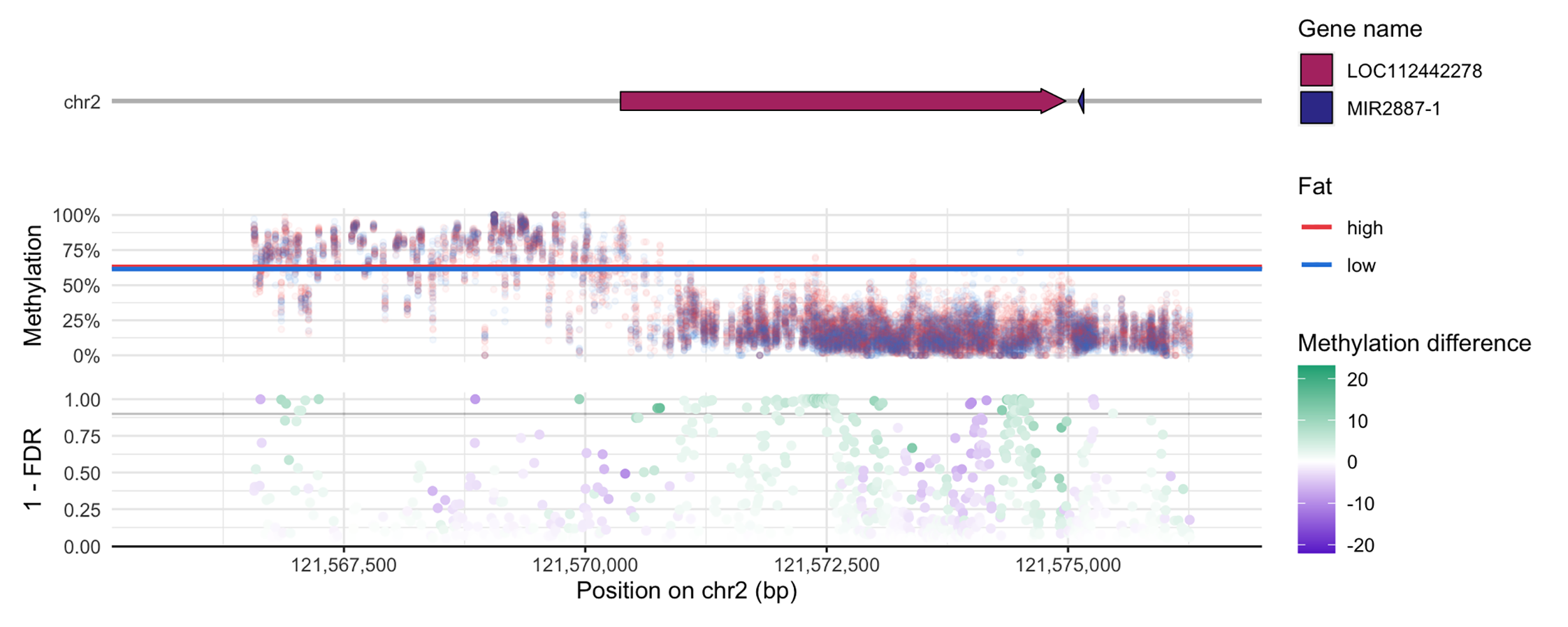

Supplement: Supplementary file 1 [file genes-12-01727-s001.zip › SupplementaryMaterial/FigureS4_DMgene_loc112332278_mir2887_1_chr2_MF.tif]
